# Antibody Seroprevalence to Spotted Fever Group Rickettsiae in Miraflores, Colombia: A Cross-Sectional Study in Humans and Dogs

**DOI:** 10.4269/ajtmh.23-0593

**Published:** 2024-04-09

**Authors:** Lídia Gual-Gonzalez, Omar Cantillo-Barraza, Myriam E. Torres, Juan C. Quintero-Vélez, Manuel Medina, Stella C. W. Self, Melissa S. Nolan

**Affiliations:** ^1^ Department of Epidemiology and Biostatistics, University of South Carolina, Columbia, South Carolina;; ^2^ Grupo Biología y Control Enfermedades Infecciosas, Universidad de Antioquia, Medellín, Colombia;; ^3^ Grupo de Investigación Epidemiología, Universidad de Antioquia, Medellín, Colombia;; ^4^ Unidad de Control de Enfermedades Transmitidas por Vectores, Secretaría de Salud de Boyacá, Tunja, Colombia

## Abstract

Tick-borne disease burdens are increasing globally, impacting mostly rural and vulnerable communities. Among the most important emerging tick-borne pathogens are the *Rickettsia* species within the spotted fever group (SFGR) because of their genetic diversity and high lethality rate. Colombia is highly affected by SFGR despite not being reportable diseases; thus, research and clinical management are neglected. Although some departments have demonstrated high seroprevalence rates, in others, such as Boyacá Department, seroprevalence is unknown. Rickettsioses have not been described in Boyacá since 1943, and conversations with local physicians raised suspicions of recent undiagnosed disease compatible with rickettsiosis in some rural areas of the department, warranting epidemiological investigation. Using biobanked human and canine samples from a previous 2021 vector-borne disease study in Miraflores municipality, Boyacá, we had an opportunity to unearth SFGR’s exposure in the region. Samples were evaluated using IgG indirect fluorescent assays against SFGR and complemented by survey questionnaires evaluating associated factors. Findings yielded first-time SFGR serological evidence in Boyacá with a 26.5% seroprevalence among dogs and a 20.4% among humans. Human and dog seroprevalences were positively associated, suggesting the presence of domestic transmission. Owning a greater number of domestic animals (prevalence ratio adjusted for all measured factors [aPR], 1.52) and living near crop fields (aPR, 7.77) were associated with an increased likelihood of household seropositivity. Our findings are consistent with the literature in Colombia, uncovering a suspected region where the disease is endemic. Future studies are warranted to continue defining high-risk areas to determine public health intervention plans.

## INTRODUCTION

Tick-borne disease burdens are increasing globally, especially in South America, where landscapes are shifting because of land use and environmental changes.^1^^–^^3^ Different tick species have been described in South America as potential *Rickettsia* vectors within the genera *Amblyomma, Rhipicephalus,* and *Haemaphysalis*.^4^ Spotted fever group rickettsiae (SFGR) comprise a genetically diverse group of bacterial species that cause a diversity of clinical symptoms ranging from asymptomatic or mild to severe disease with a high lethality rate. In South America, SFGR pathogens are commonly associated with a few principal tick species, such as *Amblyomma cajennense* s.l., *Amblyomma patinoi, Amblyomma mixtum, Amblyomma ovale, Amblyomma aureolatum, Amblyomma maculatum, Amblyomma triste, Amblyomma sculptum, Amblyomma tigrinum,* and *Rhipicephalus sanguineus,* because of their increased vectorial capacity and anthropophilic behavior.^5^^,^^6^ Beyond these principal endemic tick species, invasive tick vector importation into new ecological regions, animal migration patterns due to mass deforestation, and illegal animal trade can pose additional concerns in SFGR epidemiology.^7^^,^^8^ Broadly, public health efforts in South America are politically targeted to key vector-borne diseases (e.g., malaria, Zika virus, dengue virus, etc.), and tick-borne diseases are neglected in public health surveillance, intervention, and clinical management.^9^ Therefore, SFGR infections can emerge uninterrupted in areas without tick surveillance and control.^9^

In Colombia, tick-borne disease surveillance is not implemented at a national level, yielding a limited understanding of the SFGR burden and distribution nationally. Ecological and epidemiological investigations have revealed the presence of several SFGR species, including *Rickettsia rickettsii, Rickettsia parkeri* strain Atlantic rainforest, and *Rickettsia amblyommatis*.^10^^–^^14^ Accordingly, epidemiological studies have demonstrated SFGR serological evidence; yet these studies vary in temporality and geographic distribution, creating large SFGR knowledge gaps.^15^^–^^17^ Since the first report of *R. rickettsii* in Colombia,^18^ which was followed by over 60 years of epidemiological silence,^19^ sporadic outbreaks have been followed by reactive serosurveys.^19^^–^^21^ *Rickettsia rickettsii*, the most virulent SFGR species, is most often described as the etiologic agent, but recent studies have suggested that infections with less severe species such as *R. amblyommatis* could offer immunological protection and are likely more prevalent than originally thought.^22^ For example, Cordoba Department had previously experienced an SFGR outbreak with a 36% lethality rate,^21^ in sharp contrast to an epidemiological study in Villeta, which revealed a 40% seroprevalence with two fatalities.^19^^,^^23^ More recent outbreaks have reported two fatalities in Uramita, Antioquia, highlighting the continued need for epidemiological studies in at-risk locations.^24^ Nevertheless, SFGR serology limits the ability to determine the true bacterial species. Despite the high seroprevalence and linked lethality revealed by these historical studies, rickettsioses remain underdiagnosed in Colombia, and therefore, preventable deaths could be caused by misdiagnosis.

Boyacá Department, Colombia, lies between four departments with historical SFGR evidence, namely, Cundinamarca,^25^ Arauca,^26^ Casanare,^12^ and Antioquia^27^, and yet this department has never been formally surveyed for SFGR presence. Historical reports described some typhus group rickettsiosis outbreaks in the early 1900s until the 1940s, but no investigations or cases have been documented since, and none are from the spotted fever group.^28^ Conversations with local physicians and public health officials indicate that undiagnosed SFGR cases are occurring. Rickettsioses are not a notifiable disease in Colombia; therefore, when clinically compatible patients present for clinical management, a diagnosis of rickettsiosis is excluded because of a lack of knowledge about the disease and diagnostic testing capacity. Spotted fever group rickettsiae are viewed as a low public health priority, and no surveillance funding has been allocated to elucidate epidemiological patterns of disease. The municipality of Miraflores in Boyacá Department is particularly suspected to have undiagnosed cases given its large agricultural worker population, year-round warm temperate climate, and tick distribution (V. Beltrán, personal communication, July 18, 2022). Moreover, the municipality’s high proportion of feral dogs provides support for local peridomestic transmission cycles. The SFGR rate in the municipality of Miraflores, Colombia, is unknown, but epidemiologic risk factors are present, suggesting a high potential for its transmission locally. Therefore, the current study aims to elucidate the potential seroprevalence of human and canine SFGR using banked serum samples from an earlier cross-sectional vector-borne disease surveillance study.

## MATERIALS AND METHODS

### Study area.

**Figure 1.**
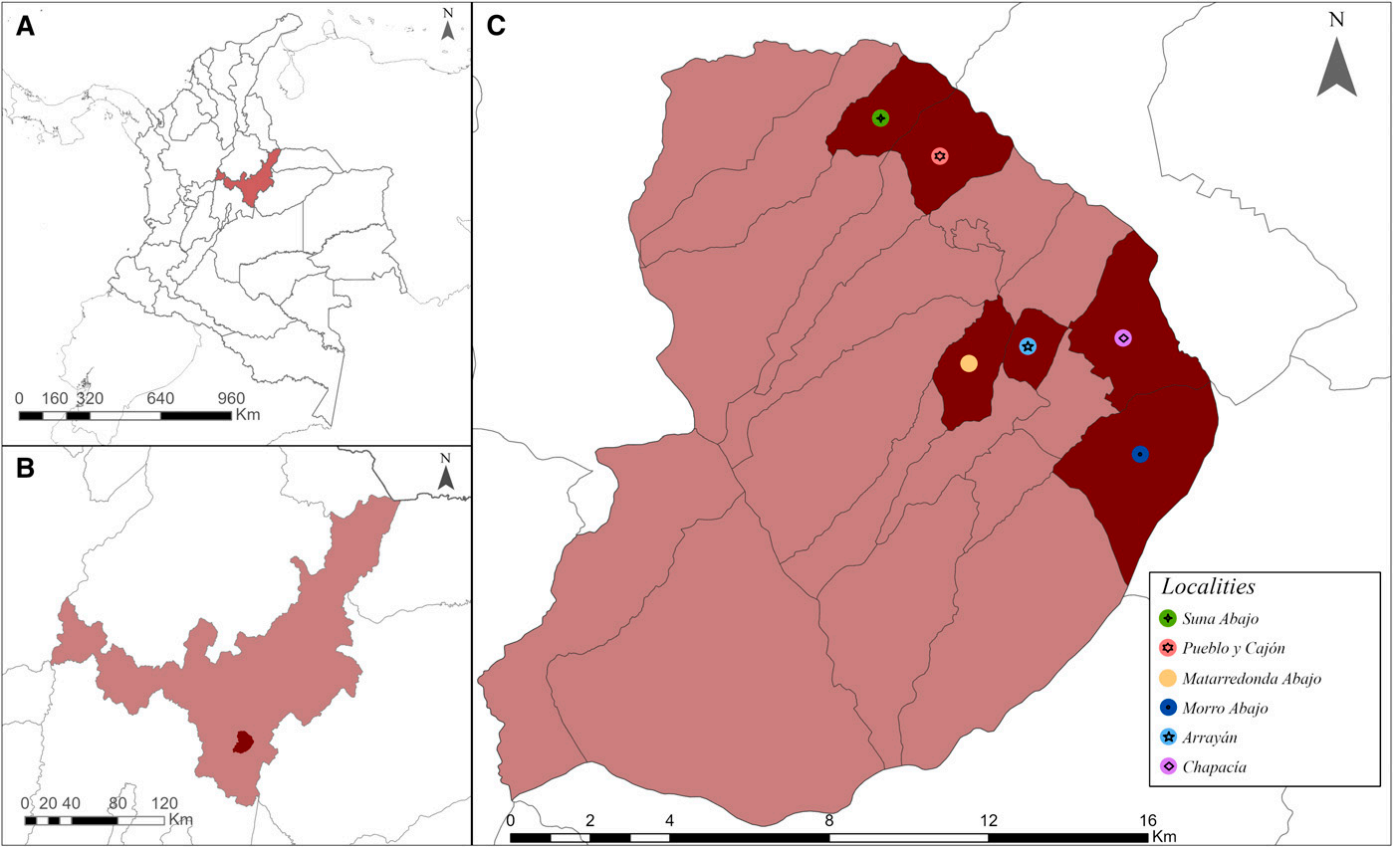
Study area and sampling localities: (**A**) Boyacá Department in Colombia; (**B**) Miraflores Municipality within Boyacá Department, (**C**) sampled localities within Miraflores Municipality (Arrayán, Chapacía, Morro Abajo, Pueblo y Cajón, Mata Redonda Abajo, and Suna Abajo).

Banked canine and human serum samples from a 2021 vector-borne disease surveillance study were available from the municipality of Miraflores, Boyacá Department, Colombia. The surveillance was performed in the rural localities of Arrayán, Chapacía, Morro Abajo, Pueblo y Cajón, Matarredonda Abajo, and Suna Abajo (Figure 1). Human subjects and ethics approval were obtained in advance, as detailed below. These six localities were selected for the original study because of their high entomological index for *Triatoma venosa*, and these home triatomine investigations surreptitiously revealed high hard tick (Acari: Ixodidae) entomologic indices, especially on domestic animals. A total of 156 human and 55 canine banked serum samples were available for the current study, and the original sampling strategy design and details are documented below. The number of banked serum samples was well powered for an SFGR serosurvey: sample size calculations based on municipality population yielded a minimum of 135 human samples and 38 dog samples, which were required to estimate a 20–50% SFGR anti-IgG seroprevalence in humans and a 30–70% seroprevalence in dogs with a 95% CI. Sample size calculations were performed with a sample size prevalence estimator using imperfect tests^29^ in Epitools (Ausvet, Fremantle, Australia).

### Sampling strategy.

In the original study, a probabilistic sampling was used to determine the number of households to sample. Using census data for each household and locality, households were selected as the sampling unit. Humans and canines were selected within each household and as the analysis unit. The original study’s inclusion criteria included persons of all ages, races/ethnicities, and sex with a permanent residence in the study area and the ability to provide informed consent. The exclusion criteria included people that had any comorbidity preventing them from providing a blood or tissue sample or anyone with a known previous diagnosis of Chagas disease. All study participants signed an informed consent allowing for their samples to be used in future infectious disease serology studies. A total of 120 households were initially approached for inclusion, and 155 individuals agreed to participate and were able to provide a plasma sample. Participating individuals with household-owned dogs were asked permission to collect a canine blood sample and to provide written informed consent to draw blood from their dog. A total of 102 participating households had owned or feral dogs on their property; of those households, 31 permitted sample collection, and a total of 55 canine samples were collected.

#### Serological samples.

Approximately 4–5 mL of peripheral whole blood was collected in ethylenediaminetetraacetic acid (EDTA) tubes through venipuncture from each participant. Samples were later centrifuged at the local hospital and stored at 4–8°C until processed in the Laboratory of Biology and Control of Infectious Diseases (BCEI) at the Universidad de Antioquia main campus in Medellín, Colombia, where samples were permanently stored at –80°C.

A trained veterinary doctor collected samples of 3–4 mL of peripheral blood from the cephalic vein in EDTA tubes. Canine blood samples followed the same processing, transport, and biobanking procedures as human samples.

#### Serological testing.

Commercial Rickettsia rickettsii indirect fluorescent antibody (IFA) substrate slides (Fullerlabs, Fullerton, CA) were used to evaluate the presence of SFGR IgG in human and canine plasma. These slides contain formalin-inactivated antigens tailored to host species in Vero 76 cells with 30–40 infected cells per field when a 40× lens was used in a fluorescence microscope. Plasma samples and a positive control were diluted in phosphate-buffered saline (PBS) at a 1:64 dilution; 10-μL volumes of diluted sample and negative and positive controls were placed in the prepared slides. These slides were incubated for 30 minutes at 37 ± 0.5°C, followed by a 10-minute PBS wash. Ten microliters of the conjugate was then placed in the slides and incubated for 30 minutes at 37 ± 0.5°C in the dark, followed by a 10-minute PBS wash. These slides were then mounted and observed under the microscope using 40× and 100× magnification. Samples showing distinctly fluorescent bacteria at a 1:64 dilution were further diluted at 1:128 titers and processed using the same steps and evaluated again under the microscope. Samples showing fluorescence bacteria at a 1:128 dilution were considered positive for IgG antibodies. Given the cross-reactivity potential between antibodies against Rickettsia rickettsii and other SFGR species, positives were defined as “SFGR exposure.”

### Evaluation of associated factors.

A survey was provided to each participating household. The questionnaire was divided into five sections, of which we used the following: 1) demographics (age of household head, sex, neighborhood; 2) household conditions, including wall, floor, and roof materials and exterior structures; 3) hygienic and lighting conditions around/inside the household; 4) presence of domestic, sylvatic, and synanthropic animals, including animal species and location of the animals with respect to the household; and 5) vegetation presence and type around the domestic environment.

## STATISTICAL ANALYSES

Point prevalence and 95% CIs of SFGR antibody were determined separately for humans and animals using the bayes.prevalence function for Bayesian estimation of prevalence developed by Diggle^30^ that accounts for test sensitivity (85–100%) and specificity (99–100%)^31^ (available at http://www.lancs.ac.uk/staff/diggle/prevalence-estimation.R/). Fisher’s exact tests and *t*-tests were used to compare the distributions of seropositive and seronegative samples across household-level survey answers for categorical and continuous variables, respectively. Given the small sample sizes for humans and canines separately, and household level evaluation of covariates, an outcome variable of “at least one seropositive human or canine” was created to determine household seropositivity. Univariable and multivariable complementary log-log regression analyses were performed to evaluate household seropositivity and the associated domestic risk factors, with random effects at the locality level to account for the probabilistic sampling strategy. Variance inflation factors were calculated for stepwise variable selection for the final model, and model fit was evaluated using the Akaike and Bayesian information criteria (AIC and BIC). All statistical analyses were performed using R Studio, R v. 4.1.1 (R Core Team, Vienna, Austria).

## RESULTS

The study included 83 households with a total of 155 human samples and 52 canine samples across six localities in the municipality of Miraflores, Boyacá Department. The first screening evaluated samples at 1:64 titers: 60 (38.7%) human samples and 29 (55.8%) canine samples showed distinctive fluorescence under the microscope. Next, 30 (19.4%) human and 13 (25.0%) canine samples were positive for *R. rickettsii* anti-IgG antibodies at 1:128 titers, and these samples were defined as SFGR seropositive. Adjusted point seroprevalence estimates and 95% CIs yielded a 26.5% (95% CI: 16.9–38.1%) seroprevalence for dogs and 20.4% (95% CI: 15.1–26.6%) for humans. No significant differences were found between demographic variables and seropositive or seronegative groups for humans and dogs (Table 1).

**Table 1:** Demographic household characteristics of seropositive and seronegative human and canine individuals in Miraflores, Boyacá

Factor	Humans			Canines		
	SFGR IgG Positive*	SFGR IgG Negative	*P*-Value^†^	SFGR IgG Positive*	SFGR IgG Negative	*P*-Value^†^
	*N* = 30 (%)	*N* = 125 (%)		*N* = 13 (%)	*N* = 39 (%)	
Sex			0.84			0.34
Female	15 (50.0)	66 (52.8)		3 (23.1)	16 (41.0)	
Male	15 (50.0)	59 (47.2)		10 (76.9)	23 (59.0)	
Age, mean ± SD	49.9 ± 21.1	49.4 ± 23.7	0.90	5.8 ± 2.8	4.8 ± 3.7	0.34
Locality			0.81			0.97
Arrayán	1 (3.3)	9 (7.2)		0	0	
Chapacía	9 (30.0)	27 (21.6)		3 (23.0)	6 (15.4)	
Pueblo y Cajón	3 (10.0)	20 (16.0)		0	0	
Suna Abajo	8 (26.7)	35 (28.0)		4 (30.8)	15 (38.5)	
Matarredonda Abajo	4 (13.3)	20 (16.0)		4 (30.8)	11 (28.2)	
Morro Abajo	5 (16.7)	14 (11.2)		2 (15.4)	15 (38.5)	
Household characteristics						
Wall material			0.99			0.35
Adobe	0	1 (0.8)		0	3 (7.7)	
Brick	20 (66.7)	82 (65.6)		6 (46.1)	21 (53.8)	
Other	10 (33.3)	40 (32.0)		6 (46.1)	15 (38.5)	
Wall finish			0.84			0.99
Completely tarnished	9 (30.0)	43 (34.4)		3 (23.0)	11 (28.2)	
Partially tarnished	8 (26.7)	43 (34.4)		4 (30.7)	17 (43.6)	
Not tarnished	2 (6.7)	8 (6.4)		0	0	
Other	1 (3.3)	3 (2.4)		0	2 (5.1)	
Roof material			0.42			0.37
Zinc	8 (26.7)	20 (16.0)		2 (15.4)	2 (5.1)	
Clay tile	0	5 (4.0)		0	3 (7.7)	
Fiber cement tile	3 (10.0)	15 (12.0)		2 (15.4)	3 (7.7)	
Other	16 (53.3)	80 (64.0)		8 (61.5)	29 (74.3)	
Floor material			0.42			0.17
Earth	2 (6.7)	5 (4.0)		2 (15.4)	2 (5.1)	
Cement	15 (50.0)	51 (40.8)		3 (23.0)	15 (38.5)	
Tiles	0	8 (6.4)		1 (7.7)	0	
Other	12 (40.0)	57 (45.6)		7 (53.8)	21 (53.8)	
Outdoor structures						
Chicken coop	25 (83.3)	105 (84.0)	0.99	12 (92.3)	32 (82.0)	0.66
Barn	11 (36.7)	47 (37.6)	0.99	6 (46.1)	16 (41.1)	0.76
Feeder	1 (3.3)	6 (4.8)	0.99	0	0	0.99
Pigsty	3 (10.0)	16 (12.8)	0.99	1 (7.7)	4 (10.2)	0.99
Hutch	3 (10.0)	6 (4.8)	0.38	0	4 (10.2)	0.56
Porch	2 (6.7)	10 (8.0)	0.99	1 (7.7)	1 (2.6)	0.44
Oven	13 (43.3)	63 (50.4)	0.55	4 (30.7)	19 (48.7)	0.34
Mill	3 (10.0)	15 (12.0)	0.99	0	3 (7.7)	0.56
Woodpile	23 (76.7)	73 (58.4)	0.09	8 (61.5)	21 (53.8)	0.75
Rockpile	5 (16.7)	21 (16.8)	0.99	3 (23.0)	8 (20.5)	0.99
Bushes present	7 (23.3)	31 (24.8)	0.99	2 (15.4)	14 (35.9)	0.29
Acceptable hygienic conditions^‡^	27 (90.0)	117 (93.6)	0.19	12 (92.3)	32 (82.0)	0.56
At least one seropositive dog in the household			**0.03**			
Yes	7 (23.3)	15 (12.0)		–	–	–
No	3 (10.0)	33 (26.4)		–	–	–
At least one seropositive human in the household						**0.03**
Yes	–	–		8 (61.5)	9 (23.1)	
No	–	–		4 (30.7)	24 (61.5)	
Animals present in the household perimeter						
Dogs	30 (100.0)	115 (92.0)	0.21	–	–	–
Chickens	27 (90.0)	100 (80.0)	0.29	11 (84.6)	28 (71.8)	0.48
Swine	9 (30.0)	24 (19.2)	0.22	2 (15.4)	3 (7.7)	0.59
Cattle	12 (40.0)	45 (36.0)	0.68	6 (46.1)	12 (30.8)	0.33
Horses	2 (6.7)	10 (8.0)	0.99	0	4 (10.2)	0.56
Cats	24 (80.0)	92 (73.6)	0.64	8 (61.5)	29 (74.3)	0.48
Domestic birds	1 (3.3)	9 (7.2)	0.69	0	0	0.99
Domestic rabbits	6 (20.0)	11 (8.8)	0.10	2 (15.4)	4 (10.2)	0.63
Sylvatic animal presence around or inside the household						
Opossums	16 (53.3)	68 (54.4)	0.99	9 (69.2)	24 (61.5)	0.75
Rodents	20 (66.7)	84 (65.6)	0.99	8 (61.5)	23 (59.0)	0.99
Bats	18 (60.0)	54 (43.2)	0.11	8 (61.5)	16 (41.0)	0.22
Pigeons	1 (3.3)	5 (4.0)	0.99	–	–	–
Wild rabbits	1 (3.3)	1 (0.8)	0.35	–	–	–
Location where the sylvatic animals have been seen						
Inside the house	2 (6.7)	11 (8.8)	0.99	1 (7.6)	3 (7.7)	0.99
Around the house	26 (86.7)	101 (80.8)	0.60	11 (84.6)	28 (71.8)	0.47
In the forest	2 (6.7)	18 (14.4)	0.37	3 (23.0)	5 (12.8)	0.39
Type of vegetation around the house						
Palm trees	2 (6.7)	0	**0.03**	–	–	–
Bushes	28 (93.3)	104 (83.2)	0.25	11 (84.6)	36 (92.3)	0.59
Trees	22 (73.3)	85 (68.0)	0.66	8 (61.5)	23 (59.0)	0.99
Epiphyte plant	16 (53.3)	74 (59.2)	0.68	7 (53.8)	22 (56.4)	0.99
Forest	12 (40.0)	47 (37.6)	0.83	8 (61.5)	20 (51.3)	0.75
Pasture	8 (26.7)	28 (22.4)	0.63	0	6 (15.4)	0.32
Crop fields	29 (96.7)	114 (91.2)	0.46	–	–	–

SFGR = spotted fever group rickettsiae. Bolding represents values with significance level of α < 0.05.

* SFGR positive is defined as having a >1:128 titer reaction on Fullerlab’s *R. rickettsia* indirect fluorescent antibody diagnostic.

^†^ Fisher’s exact test to evaluate differences between seropositive and seronegative groups for categorical variables and *t*-test to evaluate continuous variables.

^‡^ Acceptable conditions are defined as appropriate waste and garbage disposal, indoor/outdoor item arrangement, and minimal out-of-place objects.

Despite an extensive domestic environmental survey, very few statistically significant findings were noted, likely because of the generally homogeneous living conditions and cultural practices within this municipality. A univariate statistically significant association between having an SFGR-exposed human (*P =* 0.03) and canine (*P =* 0.03) was noted, suggesting domestic exposure to both species concurrently. Having palm trees around one’s home was associated with human SFGR exposure (*P =* 0.03). Having bats (*P =* 0.10) and domestic rabbits (*P =* 0.10) around one’s property neared significance with human SFGR seropositivity. Lastly, although locality was not statistically associated with SFGR seropositivity, it should be noted that Morro Abajo (26.3% SFGR seropositivity), Chapacia (25%), and Suna Abajo (18.6%) had the highest human seropositive rates.

Table 2 shows household seropositivity (having at least one seropositive human or canine) and its associated household-level factors. The presence of more domestic animal species was associated with seropositivity according to both univariate and multivariate models (prevalence ratio [PR]: 1.25; 95% CI: 1.01–1.54; and prevalence ratio adjusted for all the measured factors [aPR]: 1.52; 95% CI: 1.12–2.05), where households were more likely to have a seropositive human or canine individuals for each additional animal species within the domestic property. Moreover, having crop fields around the peridomicile was associated with seropositivity in the multivariate model (aPR: 7.77; 95% CI: 1.06–23.69), where households were more likely to have a seropositive human or canine than those without crop fields nearby.

**Table 2:** Univariable and multivariable mixed effects complementary log-log regression analyses for household SFGR IgG seropositivity

Measured Factors	PR	PR 95% CI		*P*-Value	aPR	aPR 95% CI		*P*-Value
Sex (female)	1.10	0.60	1.81	0.74	1.66	0.69	3.91	0.26
Age (years)	1.00	0.99	1.01	0.91	1.00	0.99	1.02	0.86
Number of different domestic animal species	1.25	1.01	1.54	**0.04**	1.52	1.12	2.05	**0.01**
Number of different sylvatic species	0.95	0.64	1.33	0.76	0.62	0.37	1.04	0.07
Sylvatic animal presence								
Inside the household	0.77	0.20	2.03	0.66	0.50	0.09	2.68	0.43
Around the household	0.94	0.41	1.81	0.88	1.46	0.28	6.97	0.65
In the forest	0.99	0.40	1.99	0.99	0.62	0.17	2.24	0.47
Property vegetation								
Bushes	0.80	0.32	1.62	0.60	0.67	0.17	2.60	0.56
Epiphyte plants	0.82	0.44	1.40	0.50	0.51	0.22	1.17	0.11
Forest	1.29	0.70	2.07	0.39	1.15	0.39	3.24	0.80
Crop fields	3.57	0.60	8.78	0.15	7.77	1.06	23.69	**0.04**
Pasture fields	0.74	0.35	1.40	0.38	1.17	0.40	3.27	0.78

aPR = prevalence ratio adjusted for all the measured factors included in the table; PR = prevalence ratio; SFGR = spotted fever group rickettsiae. Bolding represents values with significance level α < 0.05.

## DISCUSSION

This study presents the first serological evidence of SFGR among humans and canines in the Boyacá Department of Colombia and the associated domestic risk factors. Our results indicate notable SFGR exposure within humans (20.4% seroprevalence) and dogs (26.5% seroprevalence) in the municipality of Miraflores that, to date, has been undiagnosed. SFGR seroprevalence rates were higher in the localities of Morro Abajo, Chapacía, and Suna Abajo, which could be related to these localities’ shared ecologies, cultural dependence on domestic farming, and/or local environmental changes.^32^ Additionally, this serosurvey identified a strong relationship between SFGR seropositivity among humans and canines within the same household, providing evidence that focal transmission is the most likely infection source in this study population. Furthermore, households with a greater number of domestic animal species had higher odds of SFGR seropositivity, suggesting that dogs might not serve as the only animal host reservoir in established local transmission cycles. Overall, this study highlights that SFGR remains a neglected disease in Colombia, with up to one-fifth of Miraflores departmental residents having serologic evidence of SFGR exposure; thus, public health surveillance is warranted to mitigate future disease emergence.

This serosurvey unearthed an estimated 20.4% seroprevalence among humans in the municipality of Miraflores, Boyacá, Colombia. This finding is consistent with other national studies where the seroprevalence using a titer cutoff value of ≥1:128 has been estimated between 25.6% and 26.7%^15^^,^^16^^,^^23^; other studies, with titer cutoff values of ≥1:64 or lower, have reported 32–60% seropositivity, which makes comparison with the current study unreliable.^33^^–^^35^ Miraflores is a mountainous territory, part of the Eastern Cordillera of the Andean Mountain range. Its economy is based largely on agriculture and livestock^32^; therefore, it was not surprising to find properties with crop fields and a larger number of domestic animals associated with household seropositivity. We identified that for each additional domestic animal species within the household, there was 52% greater probability of having a seropositive member within the household. This finding is similar to that of Quintero et al,^16^ who found that the total number of animals within the household was associated with an increased likelihood of seropositivity. Similarly, human seropositivity was associated with canine seropositivity, findings consistent with the scientific literature that considers dogs an important bridge species introducing sylvatic diseases to humans and their environment.^15^^,^^16^^,^^36^

Approximately one-fourth of the dogs in this study were SFGR seropositive (26.5% seroprevalence), consistent with other national scientific literature, where canine infection ranges by ecological region. Studies in Villeta, Cundinamarca, a municipality with similar Andean mountain ecology, report dog SFGR titers at ≥1:64 in 14.4–41% of local dogs.^25^^,^^37^^,^^38^ Although these studies did not identify related risk factors, seropositivity among domestic animals was higher than among humans in the same region, consistent with our study and supported by biological plausibility that dogs experience greater tick exposure. Other studies in rural municipalities within the neighboring Antioquia Department have reported higher canine seropositivity rates (35.6–38.2%, at ≥1:128 titers)^16^^,^^39^; however, the topography, fauna, and flora in this region are notably different from those of our study location, which might explain the ecological differences contributing to lower local transmission rates.

In Boyacá Department, dogs are present as pets, working animals, or guardians, especially in rural areas. Dogs roam freely inside and outside households and are in contact with other animals, which represents a possible risk for zoonotic transmission. Dogs are known reservoirs of zoonotic infections of human importance and are more frequently infested with ticks; therefore, they become a potential source of tick-borne illness transmission in areas of high endemicity.^40^ Conversations with local farmers confirmed tick presence among their dogs, cattle, and other domestic animals in this municipality. Further, local studies demonstrate that canines are associated with human tick exposures and a higher likelihood of *Rickettsia* spp. seroprevalence in humans nationally.^15^ However, the scientific literature is mixed on the exact role canines play in SFGR transmission nationally, which highlights the need for regional and national epidemiological studies to elucidate SFGR transmission dynamics for enhanced public health interventions. Despite an unclear understanding of the epidemiological role canines play in human SFGR infection, canines are considered sentinels because of their high levels of *Rickettsia* antibodies and can be used to assess ongoing transmission in high-risk regions.^41^

Although this is the first report of human and canine exposure to SFGR in Miraflores, the emergence of novel *Rickettsia rickettsii* outbreaks was recently noted in Uramita, another Colombian region of nonendemicity with similar climate and demographic characteristics.^24^ Conversations with local physicians unveiled several incidents of suspected SFGR human cases locally, which were never properly tested because of a lack of SFGR diagnostic infrastructure. Moreover, previous documented rickettsiosis in Boyacá during the 20th century increased the suspicion of misdiagnosed SFGR locally, despite the absence of recently reported cases.^28^ Given that in other locations *R. amblyommatis* has been found to be highly prevalent among tick vectors, in comparison to pathogenic *Rickettsia* spp., the current SFGR exposure in this region could be due to nonpathogenic species, but the lack of infrastructure challenges an accurate species identification.^42^ Except for limited research by major universities across Colombia and international academic partners, the low clinical awareness and lack of national public health SFGR infrastructure propagate SFGR misdiagnosis and undertreatment nationally.^43^ To better understand the risk and severity of SFGR infections, the National Institutes of Health in Colombia should aim to develop standardized methods and protocols to improve the identification and reporting of confirmed cases, especially among patients with undifferentiated febrile illness.

There are a few study limitations worth noting. This study used banked serological samples collected a year earlier from a cross-sectional Chagas disease surveillance investigation. Therefore, the SFGR species and temporal disease course could not be verified by molecular diagnostics or a second convalescent-phase sample. As acute disease-phase patient serum samples were not available for molecular evaluation, and *Rickettsia rickettsii* cross-reacts with other SFGR pathogens by IFA, we used a conservative non-species-specific definition of SFGR to encompass the possibility that the serologic reaction might be in response to a range of pathogenic and mild disease-causing SFGR agents. To confirm true SFGR exposure, we used a conservative 1:128 titer cutoff to define SFGR positivity. Next, the original study design and questionnaire were designed for Chagas disease, which failed to capture some SFGR-specific risk factors: socioeconomic factors, outdoor and tick exposure, and history of clinical illness. Lastly, this population sample was limited to one municipality in the Boyacá Department, and therefore, the results may not be comparable to those of other regions within the department or the country.

In conclusion, this is the first serological report of SFGR in the department of Boyacá, Colombia. The findings that nearly one-fifth of residents and one-fourth of dogs have SFGR exposure are important to promote SFGR awareness among physicians and public health practitioners in the area and neighboring regions. The municipality of Miraflores is a highly rural, mostly agricultural society with high biodiversity. Human SFGR infections were associated with having seropositive dogs in the household. Moreover, domestic SFGR was associated with having a greater number of domestic animals on the property and residing adjacent to crop fields. Herein, the findings indicate possible transmission risk in the area, as most individuals culturally partake in these domestic practices. Rickettsioses remain neglected diseases in low- and middle-income countries,^9^ with SFGR studies in Colombia reduced to only a few departments. Public health efforts are warranted to better understand local transmission dynamics to prevent unnecessary morbidity and mortality from these emerging, treatable diseases.

## Data Availability

The data obtained from this study is available upon request to the corresponding author.
